# Increased Mortality of Respiratory Diseases, Including Lung Cancer, in the Area with Large Amount of Ashfall from Mount Sakurajima Volcano

**DOI:** 10.1155/2012/257831

**Published:** 2012-03-27

**Authors:** Kenta Higuchi, Chihaya Koriyama, Suminori Akiba

**Affiliations:** Department of Epidemiology and Preventive Medicine, Kagoshima University Graduate School of Medical and Dental Sciences, 8-35-1 Sakuragaoka, Kagoshima 890-8544, Japan

## Abstract

*Objectives*. Mount Sakurajima in Japan is one of the most active volcanoes in the world. This work was conducted to examine the effect of volcanic ash on the chronic respiratory disease mortality in the vicinity of Mt. Sakurajima. *Methods*. The present work examined the standardized mortality ratios (SMRs) of respiratory diseases during the period 1968–2002 in Sakurajima town and Tarumizu city, where ashfall from the volcano recorded more than 10.000 g/m^2^/yr on average in the 1980s. *Results*. The SMR of lung cancer in the Sakurajima-Tarumizu area was 1.61 (95% CI = 1.44–1.78) for men and 1.67 (95% CI = 1.39–1.95) for women while it was nearly equal to one in Kanoya city, which neighbors Tarumizu city but located at the further position from Mt. Sakurajima, and therefore has much smaller amounts of ashfall. Sakurajima-Tarumizu area had elevated SMRs for COPDs and acute respiratory diseases while Kanoya did not. *Conclusions*. Cristobalite is the most likely cause of the increased deaths from those chronic respiratory diseases since smoking is unlikely to explain the increased mortality of respiratory diseases among women since the proportion of smokers in Japanese women is less than 20%, and SPM levels in the Sakurajima-Tarumizu area were not high. Further studies seem warranted.

## 1. Introduction

**Figure 1:**
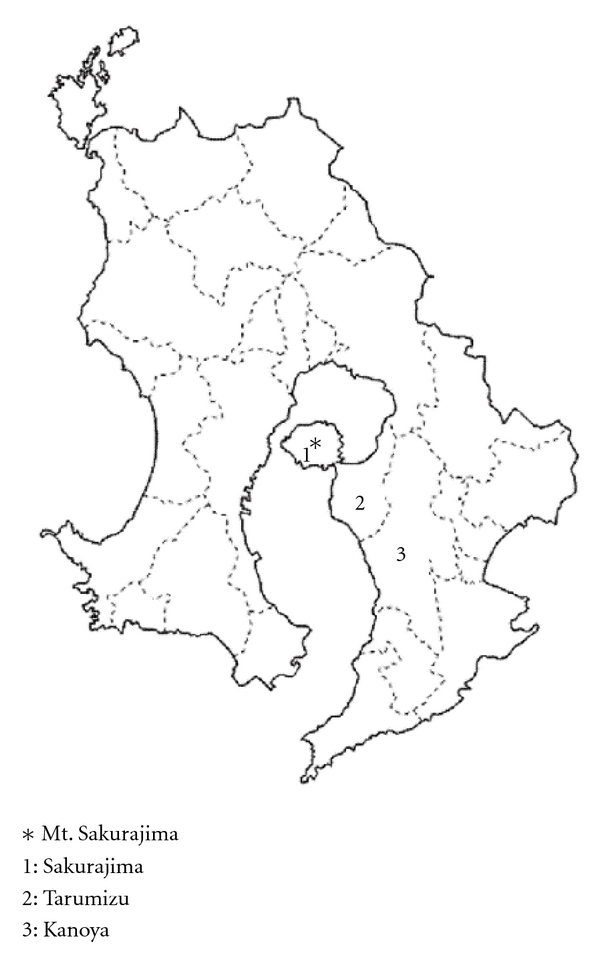
Map of Mt. Sakurajima and the study areas.

Mount Sakurajima in Kagoshima, Japan, is the world's most active volcanoes located near metropolitan areas (Figure 1). During a large eruption in 1914, lava discharged by the volcano connected the Island of Mt. Sakurajima to the Ohsumi peninsula. Since then, the volcano has become active every 10–30 years; active periods were around 1935, 1946, 1956–1967, and the period between 1972 and 2001 with its peak in 1985. In the neighborhood of Mt. Sakurajima, the largest amounts of ashfall were recorded in Sakurajima town, which is located on the foot of this mountain, and Tarumizu city, which are 5–15 km away from Mt. Sakurajima. In the 1980s, the amounts ashfall in Sakurajima-Tarumizu area exceeded 10,000 g/m^2^/yr on average according to the official report of Kagoshima Prefectural Government.

Ashes from Mt. Sakurajima contain approximately 60% of SiO_2_, which was mainly noncrystalline [1]. Recently, Hillman reported that the Sakurajima ash in general contains up to 7 wt% of cristobalite but no other silica polymorphs [2]. The presence of cristobalite in ash raises concerns about adverse health effects of long-term human exposure to ash. Crystalline silica is found in not only the ash from Mt. Sakurajima but also the ash from other volcanoes. In the case of Soufriere Hills volcano, Montserrat, the sub-10-micrometer fraction of ash generated by pyroclastic flows formed by lava dome collapse contains 10 to 24 weight percent of crystalline silica [3].

Although many studies examined the health effects of Mt. Sakurajima's volcanic activity among the residents in its neighborhood [4], no evident chronic health effects have been confirmed. However, the studies in the 1980s and the early 1990s could not examine the long-term effects of Mt. Sakurajima's volcanic activities, which peaked in the mid-1980s. In the present study, we examined the standardized mortality ratios of respiratory diseases, including lung cancer, in Sakurajima town and Tarumizu city during the period 1968–2002, and compared them with those of Kanoya city, which has much smaller amounts of ashfall than the Skurajima-Tarumizu area, in order to evaluate the long-term health effect of Mt. Sakurajima's volcanic activities.

## 2. Materials and Methods

 Sakurajima town has the population of about 4.000 and is situated on the foot of Mt. Sakurajima. Tarumizu city has the population of about 20.000 and is located within 10 km from Mt. Sakurajima. Kanoya city, with the population of about 100.000, neighbors Tarumizu city but is located at the further position from Mt. Sakurajima and therefore has much smaller amounts of ashfall. None of those municipalities has serious air pollution from traffic exhaustion. 

In the present study, we examined mortality of respiratory diseases other than tuberculosis. One of them was malignant neoplasm of trachea, bronchus, and lung (ICD9th: 162). We simply called this disease entity “lung cancer” (ICD9th: 162) in the present study. Other important respiratory diseases excluding pulmonary tuberculosis are “diseases of respiratory system” (ICD9th: 460–519). This disease category is divided into the following groups in ICD9th: acute respiratory infections (ICD9th: 460–466), other diseases of upper respiratory infection (ICD9th: 470–478), pneumonia and influenza (ICD9th: 480–487), chronic obstructive pulmonary disease and allied conditions (ICD9th: 490–496), pneumoconiosis and other lung diseases due to external agents (ICD9: 500–508), and other diseases of respiratory system (ICD9th: 510–519). In this study, we combined “acute respiratory infections” and “pneumonia and influenza” (ICD9th: 480–487) and called this category as acute respiratory diseases (ICD9th: 460–466, 480–487). On the other hand, the disease categories of “other diseases of upper respiratory infection” (ICD9th: 470–478) and “other diseases of respiratory system” (ICD9th: 510–519) were not included in our study. We concluded that they were not important for the present study because the number of deaths from those diseases was small, and they were, in a sense, an ill-defined disease category. Needless to say, we were interested in “pneumoconiosis and other lung diseases due to external agents” (ICD9th: 500–508), which may be associated with volcanic activities. However, the publication by the local health authority does not present the number of deaths for this disease category.

Standardized mortality ratios (SMRs) of deaths from lung cancer (ICD9th: 162), acute respiratory diseases (ICD9th: 460–466, 480–487), and COPDs (ICD9th: 490–496) in the Sakurajima-Tarumizu area (exposed area) and Kanoya city (control area) were calculated as a ratio between observed and expected numbers of deaths. The numbers of deaths from lung cancer (ICD9th: 162), acute respiratory diseases (ICD9th: 460–466, 480–487), and COPDs (ICD9th: 490–496) were obtained from the annual Vital Statistics Report published by the Kagoshima Prefectural Municipal Government. The expected numbers of deaths were obtained through multiplying age-gender-year-specific population numbers of Sakurajima-Tarumizu area or Kanoya city by corresponding gender-, age- (5-year category), and year-specific mortality rates for lung cancer (ICD9th: 162), acute respiratory diseases (ICD9th: 460–466, 480–487), and COPDs (ICD9th: 490–496) in Kagoshima Prefecture (population of about 1.6 million), which were obtained from the annual Vital Statistics Report published by the Kagoshima Prefectural Municipal Government. The 95% confidence intervals (95% CIs) of SMRs were obtained by the methods assuming poisson distribution [5]. The number of residents specific for gender and age (5-year category) in Sakurajima-Tarumizu area and Kanoya city was obtained by the national census conducted every 5 years. The population in 1970 was used for 1968–1972, 1975 for 973–1977, 1980 for 1978–1982, 1985 for 1983–1987, 1990 for 1988–1992, 1995 for 1993–1997, and 2000 for 1998–2002.

 The data on the amount of ashfall were obtained from the website of Kagoshima Prefectural Government.

## 3. Results

 The SMR of lung cancer in Sakurajima-Tarumizu area was 1.61 (95% CI = 1.44–1.78) for men and 1.67 (95% CI = 1.39–1.95) for women, indicating that lung cancer mortality was significantly increased when compared to the entire Kagoshima Prefecture. Sakurajima-Tarumizu area had a significantly increased COPD mortalities (SMR for men and women combined = 1.81, 95% CI = 1.63–2.00; data not shown in Table 1) and acute respiratory diseases (SMR = 1.12, 95% CI = 1.08–1.16). It should be noted that the SMRs for acute respiratory diseases were evidently lower than those for lung cancer and COPDs, and the differences between acute respiratory diseases and the other two diseases were significant since the 95% CI of the SMR of acute respiratory diseases did not overlap with those with the other two respiratory diseases.

Kanoya city, which is about 30 km away from Mt. Sakurajima, has ashfall of 640 g/m^2^/yr, which is less than 10% of that in Sakurajima-Tarumizu area. However, the mortality rates of lung cancer, COPDs, and acute respiratory diseases in Kanoya city were significantly decreased when compared to Kagoshima prefecture. Note that the upper 95%CIs of the SMRs for those respiratory diseases in Kanoya were below the unity.

## 4. Discussion

 The present study showed that residents in Sakurajima-Tarumizu area, located near Mt. Sakurajima, experienced relatively high mortality of respiratory diseases, including lung cancer and COPDs. The consistently high SMR of lung cancer in Tarumizu during the entire period of our statistical analysis (1968–2002) suggests long-term effects of Mt. Sakurajima, which has become active every 10–30 years over the last 100 years.

Confounding of smoking is unlikely among women since the proportion of female smokers is less than 20% in Japan [6]. The proportion of smokers among men and women was reported to be 39% and 6%, respectively, in Tarumizu city, and was 40% and 8%, respectively in Kanoya city (unpublished data provided by Kanoya Public Health center). It should also be noted that the relative risk comparing smokers and nonsmokers among women is at maximum 5 [7] since heavy smokers are rare and the age starting smoking is around 30 years of age among women [6]. The mortality of smoking-related cancers other than lung cancer in the Sakurajima-Tarumizu area among men was similar to that in Kanoya city (data not shown). Taken together, smoking is unlikely to explain the increased lung cancer risk among men and women in Sakurajima-Tarumizu area.

Suspended particulate matter (SPM) is a possible factor that increased the mortality of lung cancer and COPDs in Sakurajima-Tarumizu area. However, Nishii et al. reported the lack of significant correlation between SPM concentrations and the amount of ashfall [8]. At the observatory stations in Sakurajima town (the period of 1990–2007), Tarumizu city (the period of 1979–1993), and Kanoya city (the period of 1988–1993), the annual average concentration of SPM was 0.022–0.044 mg/m^3^, 0.025–0.035 mg/m^3^ and 0.027–0.036 mg/m^3^, respectively (Kagoshima Prefecture Government).

On the basis of what was reported by Steenland et al. [9], we estimated that the cumulative exposure of 400 mg m^−3^ year increases lung cancer risk by 1.2-folds. In the Sakurajima-Tarumizu area, the cumulative amount of ashfall in the 1980s and 1990s was nearly 200,000 g m^−2^. Since the ash of Sakurajima contains approximately 7% of cristobalite, and its 10% is assumed to be respirable material (<4 um) based on the finding of Hillman [2], cumulative exposure during that 20-year period could be as high as 1400 g m^−3^ year (=200.000 × 7% × 10%). The exposure to only 0.1% of 1400 g can increase lung cancer risk by 1.7-folds.

Cristalline silica is known to cause silicosis or pneumoconiosis [10]. However, data on silicosis were not available in the present study. In a study of national health insurance claim, a few patients were found to have diagnosed as having pneumoconiosis in the Ushine and Kaikata districts of Tarumizu, which is a heavy ashfall area [11]. Unfortunately, however, the occupational history of those cases was not available. If silicosis may not be markedly increased in the vicinity of Mt. Sakurajima, we have to assume that crystobalite is able to increase lung cancer not through silicosis. Whether or not excessive lung cancer cases occur exclusively among subjects with silicosis remains uncertain [12]. The association of silica exposure with lung cancer risk is generally, but not uniformly, stronger among silicotics than nonsilicotics. There are a couple of studies that showed an increase of lung cancer risk among nonsilicotic subjects [12]. Checkoway and Franzblau concluded that population-based or individually based risk assessments should treat silicosis and lung cancer as distinct entities whose cause/effect relations are not necessarily linked until more conclusive epidemiologic findings become available [12]. What was reported in the present study supports the notion. Having said that, it should be pointed out that cristobalite may not be the only factor involved in the increased mortality of lung cancer and COPDs since volcanic activities release various carcinogens, including radioactive materials, into the air.

The present study has several limitations. One is the lack of information on silicosis. Therefore, whether lung cancer patients had silicosis or not is not known. In addition, the mortality of silicosis and related diseases cannot be examined because the information is not made public. However, the study of Wakisaka et al. [11] suggested that silicosis is rare in the study area. In our preliminary analysis using the data for 1968–1995 in Tarumizu, the mortality of silicosis was increased by 1.6-folds among men but not increased among women. Another drawback is the lack of information of the residential history and the hours the residents stayed inside home. Hours spend outside the study areas were not available, either. It should be noted, however, that women tend to stay longer in the houses and tend not to work outside the study area. Since the increase of mortality from lung cancer and COPDs was observed not only among men but also among women, local environmental exposure is more likely to be related to the increased mortality of those diseases. The lack of information on smoking is also a serious drawback. However, as we discussed before, it is difficult for smoking to explain what was observed in the present study.

In conclusion, cristobalite is the most likely cause of the increased deaths from those chronic respiratory diseases since smoking is unlikely to explain the increased mortality of respiratory diseases among women since the proportion of smokers in Japanese women is less than 20%, and SPM levels in the Sakurajima-Tarumizu area were not high. Further studies on the health effects of Mt. Sakurajima's volcanic activities seem warranted. Those studies should include the examination of indoor levels of crystalline silica.

We selected Kanoya city as a control area since the ashfall was much less than Sakurajima-Tarumizu area while those two areas are closely located. Kanoya is slightly more urban than Sakurajima-Tarumizu. Therefore, we were afraid that the using Kanoya as a control may end up with underestimating the effects of volcanic activities on chronic respiratory disease mortality in Sakurajima-Tarumizu area. However, contrary to our expectation, Kanoya city had the mortality of respiratory disease mortality less than the entire Kagoshima, making the comparison more favorable for Sakurajima-Tarumizu area.

## 5. Conclusions

Mt. Sakurajima in Japan is one of the most active volcanoes in the world. In the last century, the volcano became active every 10–30 years. Its volcanic ash contains up to 7 wt.% of cristobalite, which is known to be carcinogenic. However, the chronic health effects of Mt. Sakurajima's volcanic activities were not clear.

This study showed that Mt. Sakurajima's volcanic activities increased the mortality of lung cancer and COPDs in the Sakurajima-Tarumizu area, which has the largest amount of ashfall from the volcano in its vicinity.

Further studies should be conducted to confirm the association between the volcanic activities and chronic respiratory disease mortality. Those studies should include the examination of indoor levels of crystalline silica.

## Figures and Tables

**Table 1:** SMR of respiratory diseases during the period 1968–2002.

	Sakurajima + Tarumizu				Kanoya			
	(high ashfall area)				(low ashfall area)			
	O*	E*	SMR	95% CI	O*	E*	SMR	95% CI
Lung cancer (ICD 9th: 162)								
Males	353	219.4	1.61	1.44–1.78	544	628.4	0.87	0.79–0.94
Females	138	82.4	1.67	1.39–1.95	165	222.0	0.74	0.63–0.86

COPDs (ICD 9th: 490–496)								
Males	252	140.3	1.80	1.68–1.91	267	375.6	0.71	0.63–0.80
Females	134	72.5	1.85	1.53–2.16	132	201.6	0.65	0.54–0.77

Acute respiratory diseases (ICD 9th: 460–466, 480–487)								
Males	389	331.1	1.17	1.06–1.29	845	945.8	0.89	0.83–0.95
Females	339	320.9	1.06	0.95–1.17	721	854.6	0.84	0.78–0.91

*Observed and expected numbers of death.
